# Polyphyllin I Inhibits *Propionibacterium acnes*-Induced Inflammation *In Vitro*

**DOI:** 10.1007/s10753-018-0870-z

**Published:** 2018-08-18

**Authors:** Tingting Zhu, Wenjuan Wu, Shuyun Yang, Donglin Li, Dongjie Sun, Li He

**Affiliations:** Department of Dermatology, First Affiliated Hospital of Kunming Medical University, Kunming, 650032 China

**Keywords:** *P. acnes*, TLR2, NF-κB, MAPK, polyphyllin I

## Abstract

*Propionibacterium acnes* (*P. acnes*) has been implicated in the progression of acne inflammation. Because current acne medications have various side effects, it is necessary to explore alternative medications possessing anti-inflammatory activity against *P. acnes*. We investigated the inhibitory effects of polyphyllin I (PPI) on *P. acnes*-induced inflammation *in vitro*. In this study, we examined the effects of PPI on the production of inflammatory cytokines in HaCaT keratinocytes treated with heat-killed *P. acnes*. These treated HaCaT keratinocytes showed increased expression of Toll-like receptor 2 (TLR2) and production of inflammatory cytokines. PPI significantly suppressed the secretion of inflammatory cytokines, including interleukin (IL)-6, IL-8, and tumor necrosis factor (TNF)-α, and the expression of TLR2 in *P. acnes*-treated cells. Moreover, we studied the influence of PPI on the nuclear factor-κB (NF-κB) and mitogen-activated protein kinase (MAPK) signaling pathways in *P. acnes*-treated keratinocytes. PPI diminished the activation of NF-κB. Phosphorylated p38 levels were markedly increased after treatment with heat-killed *P. acnes* but were decreased after treatment with PPI, while the effect of PPI on ERK phosphorylation was not significant. Heat-killed *P. acnes* and PPI did not have any effect on JNK phosphorylation. Furthermore, we confirmed that NF-κB p65 inhibitor (BAY11-7082), p38 MAPK inhibitor (SB203580), and PPI blocked the expression of IL-8 in heat-killed *P. acnes*-treated cells. These results demonstrated that PPI has potential for development as a treatment for acne inflammation.

**Electronic supplementary material:**

The online version of this article (10.1007/s10753-018-0870-z) contains supplementary material, which is available to authorized users.

## INTRODUCTION

Acne vulgaris is the 8th most common disease in the world, affecting more than 80% of individuals at some time during their lives [5, 9]. Although this is not a fatal disease, inflammatory lesions can persist for a long time, ultimately resulting in permanent scarring on the face of those with severe cases and causing serious physical and psychosocial morbidities [23]. Four factors have been identified to play critical roles in the pathogenesis of acne: sebum hyperproduction, altered follicular keratinization, *Propionibacterium acnes* (*P. acnes*) colonization and proliferation, and immune reaction and inflammation [15]. At present, it is widely accepted that acne is an inflammatory disease, inflammation is involved in all acne-related processes, and inflammatory events are involved in initiating acne lesions [3, 13, 22].

*P. acnes*, a common Gram-positive anaerobic bacterium, is part of the normal skin flora and plays an important role in acne pathogenesis. *P. acnes* stimulates the production of various inflammatory cytokines through Toll-like receptor (TLR) 2 and, to a lesser extent, TLR4, contributing to acne inflammation [14, 17]. Furthermore, *P. acnes*-activated TLR2 promotes the activation of the MAPK and NF-κB signaling pathways, which are responsible for inflammatory cytokine production and the innate immune response [11]. Therefore, the *P. acnes*-induced inflammatory response participates in acne pathogenesis, at least in part due to the activation of TLR2 and its downstream signaling pathways, namely, the MAPK and NF-κB pathways.

Antibiotics and isotretinoin are frequently used therapeutic agents for acne inflammation, but they have potentially serious detrimental effects [27]. Therefore, there is an increasing need to identify new anti-acne ingredients, especially natural products with relative safety. *Paris polyphylla* has been widely used as a natural medicine in the treatment of infectious and inflammatory diseases and of cancer in traditional Chinese medicine for approximately 2000 years [26]. In 2015, *Paris polyphylla* was officially documented as an anti-inflammatory and hemostatic agent in the Chinese Pharmacopoeia [26]. Polyphyllin I (PPI), a major steroidal saponin extracted from *Paris polyphylla* rhizomes, displays proapoptotic and anti-tumor effects [1, 4, 7]. However, no studies have shown the role and underlying mechanism of PPI-mediated anti-inflammatory activity. We aimed to evaluate the effects of PPI in *P. acnes*-induced inflammation *in vitro*.

## MATERIALS AND METHODS

### Materials

The strain of *P. acnes* (ATCC6919, Xiangfu Biotech, Shanghai, China) was obtained from the American Type Culture Collection. The bacteria were cultured in brain heart infusion (BHI) broth (Rishui Biotechnology, Qingdao, China) under anaerobic conditions. The HaCaT cell line was purchased from the cell bank of the Type Culture Collection of the Chinese Academy of Sciences (Shanghai). Cells were grown in RPMI 1640 medium (Gibco BRL, NY) supplemented with 10% heat-inactivated fetal bovine serum (FBS) (Gibco), streptomycin (100 μg/ml), and penicillin (100 U/ml) at 37 °C in a humidified atmosphere with 5% CO_2_. PPI was purchased from the Institute for Drug Control (Shanghai, China, #111590) and dissolved in dimethyl sulfoxide (DMSO). BAY11-7082 and SB203580 were purchased from Sigma (St. Louis, MO, USA).

### Preparation of *P. acnes*

*P. acnes* was cultured in the exponential phase for 2 days and in the stationary phase for 3 days. The bacteria were harvested by centrifugation at 2500 rpm for 5 min at 4 °C. The bacterial pallets were washed in cold phosphate-buffered saline (PBS) and centrifuged three times. Finally, the *P. acnes* pellet was resuspended in PBS. To obtain heat-killed bacteria, the bacterial suspension was heated at 70 °C for 30 min, and the supernatant was removed by centrifugation at 10,000 rpm for 5 min at 4 °C. This processed pellet was used for subsequent experiments.

### Cell Viability Assay

The effects of PPI on HaCaT cell viability were determined by the Cell Counting Kit-8 (CCK-8 assay: Dojindo Laboratories, Japan). CCK-8 assays were used to assess the rate of cellular proliferation and to quantify cell viability. In brief, HaCaT cells were seeded in 96-well plates with 100 μl of medium at a density of 2 × 10^5^ cells/well. After the cells were incubated with different concentrations of PPI (0, 0.3, 0.6, 0.9, and 1.2 μg/ml), 10 μl of CCK-8 solution was added to each well, and the plates were incubated for 1 h at 37 °C. Finally, we determined the optical density (OD) at 450 nm using a Microplate Reader (BioTek, USA). All experiments were conducted in triplicate.

### Enzyme-Linked Immunosorbent Assay

Cultured HaCaT cells were challenged with *P. acnes* (ATCC6919) at 0, 1.0 × 10^5^, 1.0 × 10^6^, and 1 × 10^7^ CFU. The following cytokines were determined: IL-6, IL-8, and TNF-α. An enzyme-linked immunosorbent assay (ELISA) kit (RD Systems, Minneapolis, MN) for each cytokine was used to determine the expression level according to the manufacturer’s instructions. As described in previous studies, HaCaT cells were seeded in 96-well plates at a density of 2 × 10^5^ cells/well in FBS-free medium and pretreated with different concentrations of PPI (0, 0.3, 0.6, and 0.9 μg/ml) for 2 h, followed by stimulation with heat-killed *P. acnes* (1 × 10^7^ CFU/ml) for 24 h. Cell-free supernatants were analyzed by ELISA for IL-6, IL-8, and TNF-α. In addition, the cells were pretreated with DMSO, PPI (0.9 μg/ml), or SB203580 (20 μmol) for 2 h, followed by stimulation with heat-killed *P. acnes* (1 × 10^7^ CFU/ml) for 24 h. Cell-free supernatants were analyzed for IL-8. All experiments were performed three independent times.

### Quantitative Real-Time Polymerase Chain Reaction

HaCaT cells were adjusted to a density of 2 × 10^5^ cells/well in serum-free medium and seeded in 6-well plates. Cells were pretreated with different concentrations of PPI (0.3, 0.6, and 0.9 μg/ml) for 2 h. Next, cells were stimulated with heat-killed *P. acnes* for 8 h, followed by harvesting and rinsing. The control group was incubated without PPI or bacteria. Total RNA was isolated from cells using an RNA extraction kit following the manufacturer’s instructions and quantified with a spectrophotometer at 260 nm. A reverse-transcription kit (Takara, Shiga, Japan) was used to synthesize cDNA. qRT-PCR was conducted as follows: initial denaturation at 95 °C for 30 s, followed by 40 cycles of 95 °C for 5 s, 60 °C for 30 s, and 72 °C for 60 s. The primer sets for TLR2, IL-1α, keratin 16 (K16), and GAPDH were purchased as QuantiTect primer assays (Sangon Biotech, Shanghai). GAPDH was used as an endogenous control.

### Western Blot Analysis

Cells were harvested, and total protein was extracted from each sample, separated by 10% SDS-PAGE, and then transferred to a polyvinylidene difluoride (PVDF) membrane. After being blocked in 5% skim milk for 2 h at room temperature, the membranes were incubated overnight at 4 °C with primary antibodies [K16 mouse antibody (Santa Cruz Biotechnology, Santa Cruz, CA); IL-1α mouse antibody (R&D Systems, Minneapolis, MN); TLR2 rabbit antibody (Abcam, Massachusetts, UK); and p-38, p-p38, ERK, p-ERK, JNK, p-JNK, IκBα, p-IκBα, NF-κB p65, p-NF-κB p65, and GAPDH antibodies (Cell Signaling Technology, Inc., MA, USA)]. The membranes were then washed in TBST and incubated with HRP-conjugated secondary IgG antibody at room temperature for 1 h. The bands were visualized by an ECL kit and exposed with an Amersham Imager (General Electric Company, USA). The data represent three independent experiments.

### Immunofluorescence Assay

HaCaT cells treated or untreated with PPI, stimulated or unstimulated with *P. acnes*, were harvested and rinsed. Next, cells were washed with 0.01 M phosphate-buffered saline (PBS) and fixed in 4% paraformaldehyde at room temperature for 30 min. After being permeabilized with a penetrating agent (0.2% Triton X-100) for 20 min, and blocked with 5% BSA for 1 h, cells were blocked with PBS containing 5% bovine serum albumin for 1 h at room temperature and then resuspended in 100 μl of diluted NF-κBp65 antibody (1:200, Cell Signaling Technology, Inc., MA, USA), and incubated overnight at 4 °C. Cells were rinsed and incubated with fluorescein isothiocyanate-labeled goat anti-rabbit secondary antibody (1:1000, Cell Signaling Technology, Inc., MA, USA) for 1 h in the dark, followed by staining with DAPI for 3 min. Finally, these slides were sealed by anti-fluorescence mounting medium and observed under confocal laser fluorescence microscope (Carl Zeiss Zen 2008, Carl Zeiss Inc., Germany).

### Flow Cytometry Detection

A suspension of 10^6^ cultured HaCaT cells stimulated or unstimulated with *P. acnes* was prepared and permeabilized to detect TLR2 and TLR4 protein expression. The cells were collected and washed twice with PBS, then stained with 5 ml purified anti-human TLR2 and TLR4 antibody (Abcam, Massachusetts, UK) at 4 °C for 30 min protected from light. After washing twice with PBS, the cells were collected by low-speed centrifugation (1500 r, 10 min) and incubated with 2 μl PE-conjugated goat anti-rabbit IgG mAb (Abcam, Massachusetts, UK) at 4 °C for 30 min in the dark, followed by two washes with PBS. Finally, the stained cells were suspended in 500 ml PBS, and analyzed by flow cytometry. The negative control was processed in the same way but without the anti-TLR2 and TLR4 antibodies.

### Statistical Analysis

Statistical analysis was performed using Statistical Package for Social Sciences (SPSS, version 19, IBM, Armonk, NY, USA). The results are presented as the means ± standard deviations. Differences among groups of data were analyzed using one-way analysis of variance (ANOVA). A value of *P* < 0.05 indicated statistical significance.

## RESULTS

### *P. acnes* Induced the Secretion of Inflammatory Cytokines and the Expression of TLR2 in HaCaT Cells

**Fig. 1:**
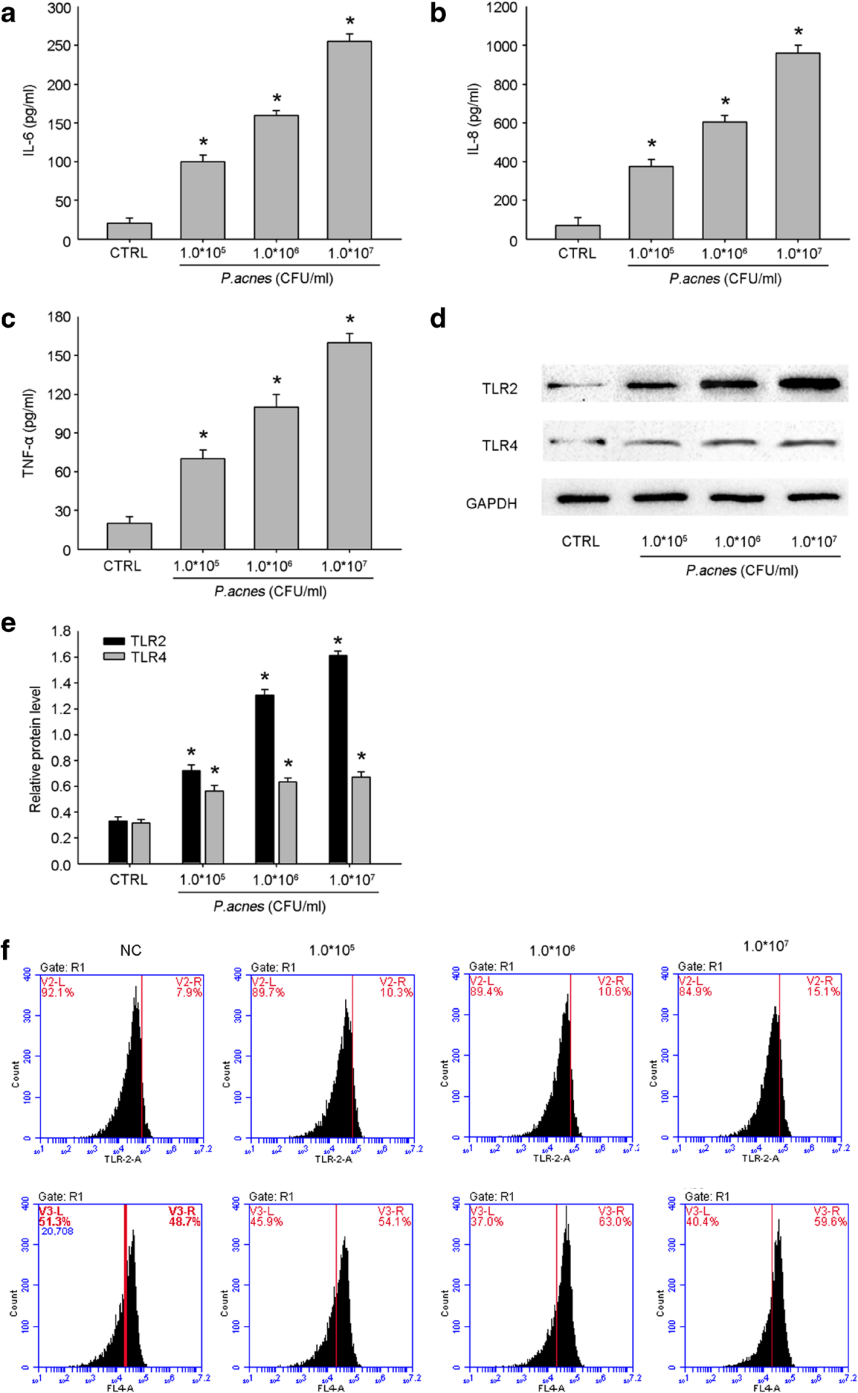
Heat-killed *P. acnes* induced inflammatory cytokine production in HaCaT cells. HaCaT cells were treated with heat-killed *P. acnes.* ELISA results with culture medium show that IL-6, IL-8, and TNF-α were increased by heat-killed *P. acnes* (**a**–**c**).Western blot analysis and flow cytometry demonstrate that TLR2 and 4 were increased by heat-killed *P. acnes* (**d**–**f**). Results are expressed by mean ± SE of three independent determinations. *P < 0.05 compared with the control group.

We first verified that *P. acnes* induces the secretion of inflammatory cytokines. The levels of inflammatory cytokines released by *P. acnes*-stimulated HaCaT cells were detected by ELISA. Untreated HaCaT cells secreted low levels of IL-6, IL-8, and TNF-α, while treatment with heat-killed *P. acnes* significantly upregulated the levels of these cytokines in a concentration-dependent manner (Fig. 1a–c). In particular, IL-8 expression levels were markedly increased. Several papers have reported that *P. acnes* may induce an inflammatory response *via* the activation of TLR2 and TLR4, which are expressed on the surface of HaCaT keratinocytes [14, 17]. Consistent with the results of previous studies, TLR2 expression levels were significantly increased following *P. acnes* stimulation (Fig. 1d–f).

### PPI Reduced the Secretion of Inflammatory Cytokines by *P. acnes*-Treated HaCaT Cells

**Fig. 2:**
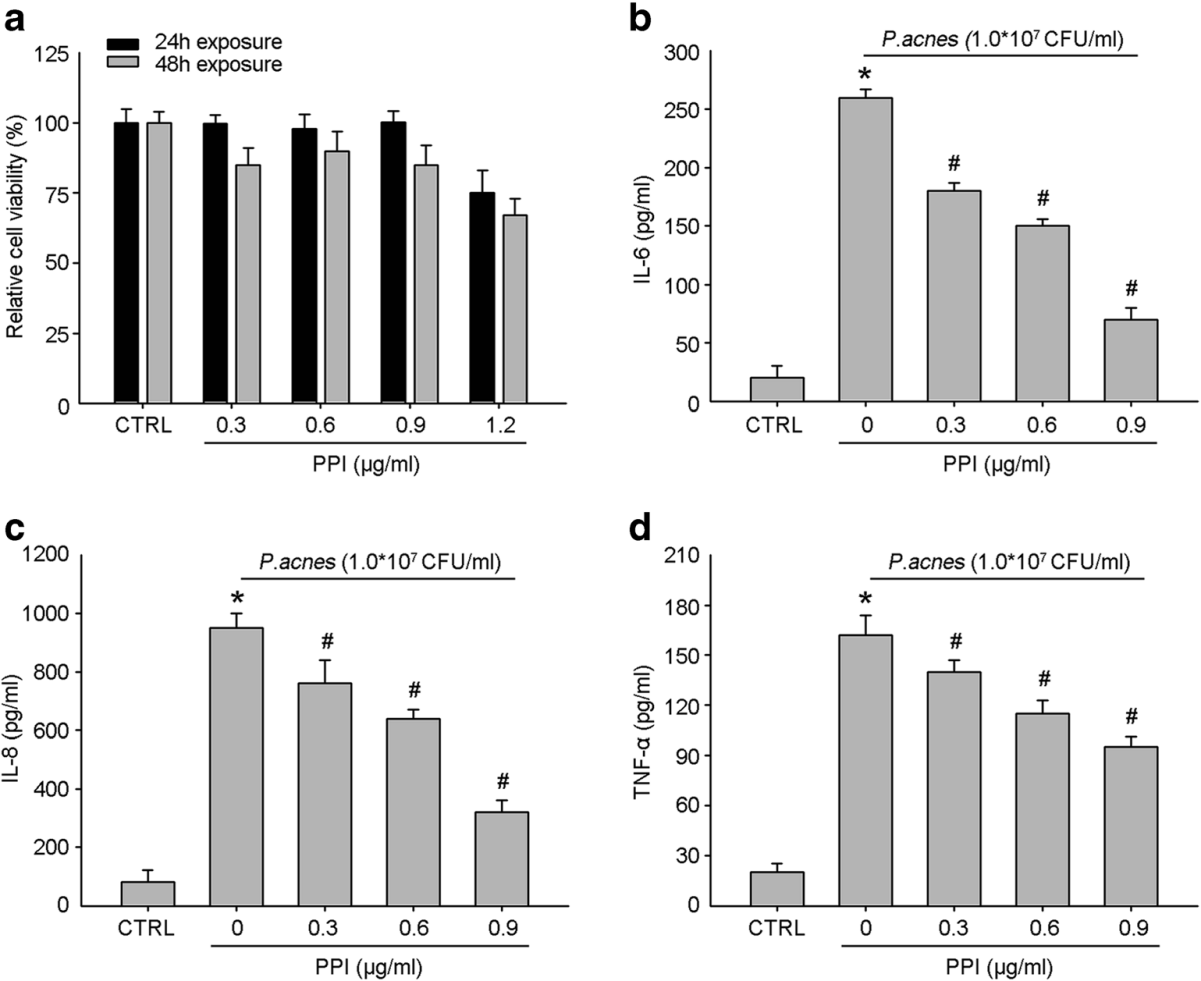
Effects of PPI on viabilities of HaCaT cells and the production of inflammatory cytokines. (**a**) Cells were exposed to PPI (at 0–1.2 μg/ml concentration) for 24 (black bars) and 48 h (gray bars). HaCaT cells were co-incubated with different concentrations (0.3, 0.6, 0.9 μg/ml) of PPI for 2 h, and then stimulated with heat-killed *P. acnes* for 24 h. The culture supernatants were subsequently isolated and analyzed for IL-6 (**b**), IL-8 (**c**), and TNF-α (**d**) production. A control group without *P. acnes* stimulation was conducted in paralleled. Data are the mean ± SE (*n* = 3). *P < 0.05 between control and each *P. acnes*-stimulated group, #*P* < 0.05 between the *P. acnes*-treated group, PPI non-treated group, and each PPI-treated group.

We investigated the toxicity of PPI at different concentrations (0.3, 0.6, 0.9, and 1.2 μg/ml) and time points (24 and 48 h) in CCK-8 assays to choose a suitable concentration range and time point for subsequent experiments. The results are shown in Fig. 2a. After 24 and 48 h, PPI exhibited no toxicity against HaCaT keratinocyte viability at a concentration of 0.3–0.9 μg/ml. We tested the antibacterial activity of PPI in preliminary experiments. The MIC of PPI was 0.625 mg/ml, while the maximum tested concentration of PPI in the current study was 0.9 μg/ml. Therefore, PPI showed little effect on the growth of *P. acnes* at this concentration. On the basis of these results, concentrations of 0.3, 0.6, and 0.9 μg/ml PPI were used in subsequent experiments. We examined the effects of PPI on the secretion of inflammatory cytokines by HaCaT cells treated with *P. acnes*. Supporting its potential anti-inflammatory effects, PPI decreased the expression levels of IL-6, IL-8, and TNF-α in a dose-dependent manner (Fig. 2b–d).

### PPI Decreased the Transcription and Expression of TLR2 in *P. acnes*-Treated HaCaT Cells and Potentially Modulated the Dyskeratosis of Epidermal Keratinocytes

**Fig. 3:**
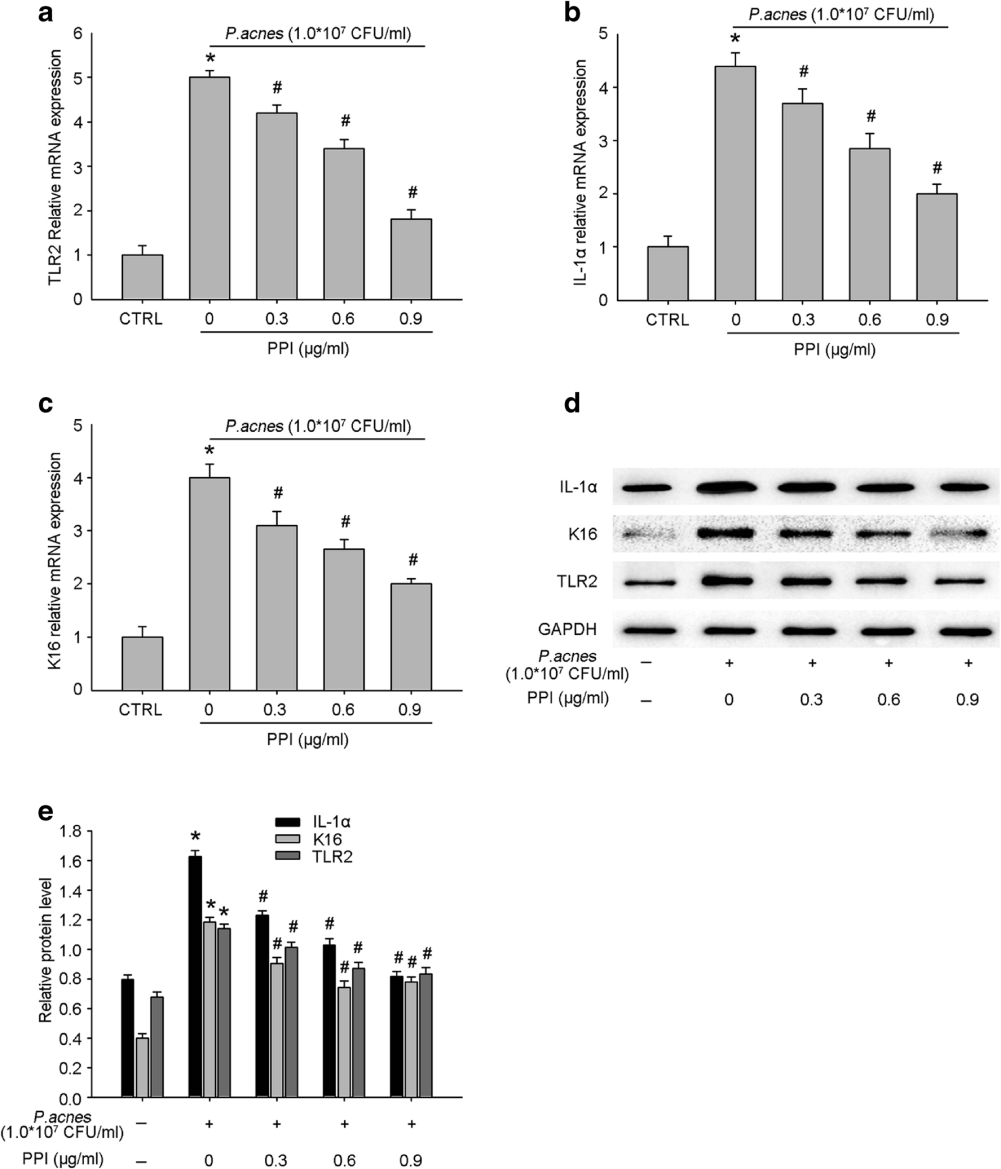
Effects of PPI on the production expression level of TLR2, IL-1α, and K16. Cells were co-incubated with DMSO or different concentrations of PPI for 2 h and then stimulated by heat-killed *P. acnes* for 8 h. A control group without *P. acnes* stimulation was done in parallel. The expression levels of TLR2 (**a**), IL-1α (**b**), and K16 mRNA (**c**) were determined as qRT-PCR. Western blotting of TLR2, IL-1α, and K16 were performed (**d**, **e**). Data are the mean ± SE (*n* = 3). *P < 0.05 between control and each *P. acnes*-stimulated group, #*P* < 0.05 between the *P. acnes*-treated group, PPI non-treated group, and each PPI-treated group.

In accordance with our previous findings, we observed that TLR2 was activated in HaCaT keratinocytes following *P. acnes* exposure, but PPI significantly reduced TLR2 mRNA and protein expression in a concentration-dependent manner (Fig. 3a–e). In addition, PPI significantly downregulated the mRNA and protein expression of IL-1α (Fig. 3b–e), a strong stimulator of follicular hyperkeratosis, and inhibited the expression of K16 (Fig. 3c–e), a marker of epidermal proliferation and abnormal differentiation, in heat-killed *P. acnes*-treated HaCaT keratinocytes. Follicular epidermal dyskeratosis is one of the major triggers of acne pathogenesis. We examined the possible beneficial effects of PPI on the hyperproliferation and abnormal differentiation of *P. acnes*-treated HaCaT cells.

### PPI Inhibited the Activation of the NF-κB and MAPK Signaling Pathways in *P. acnes*-Treated HaCaT Cells

**Fig. 4:**
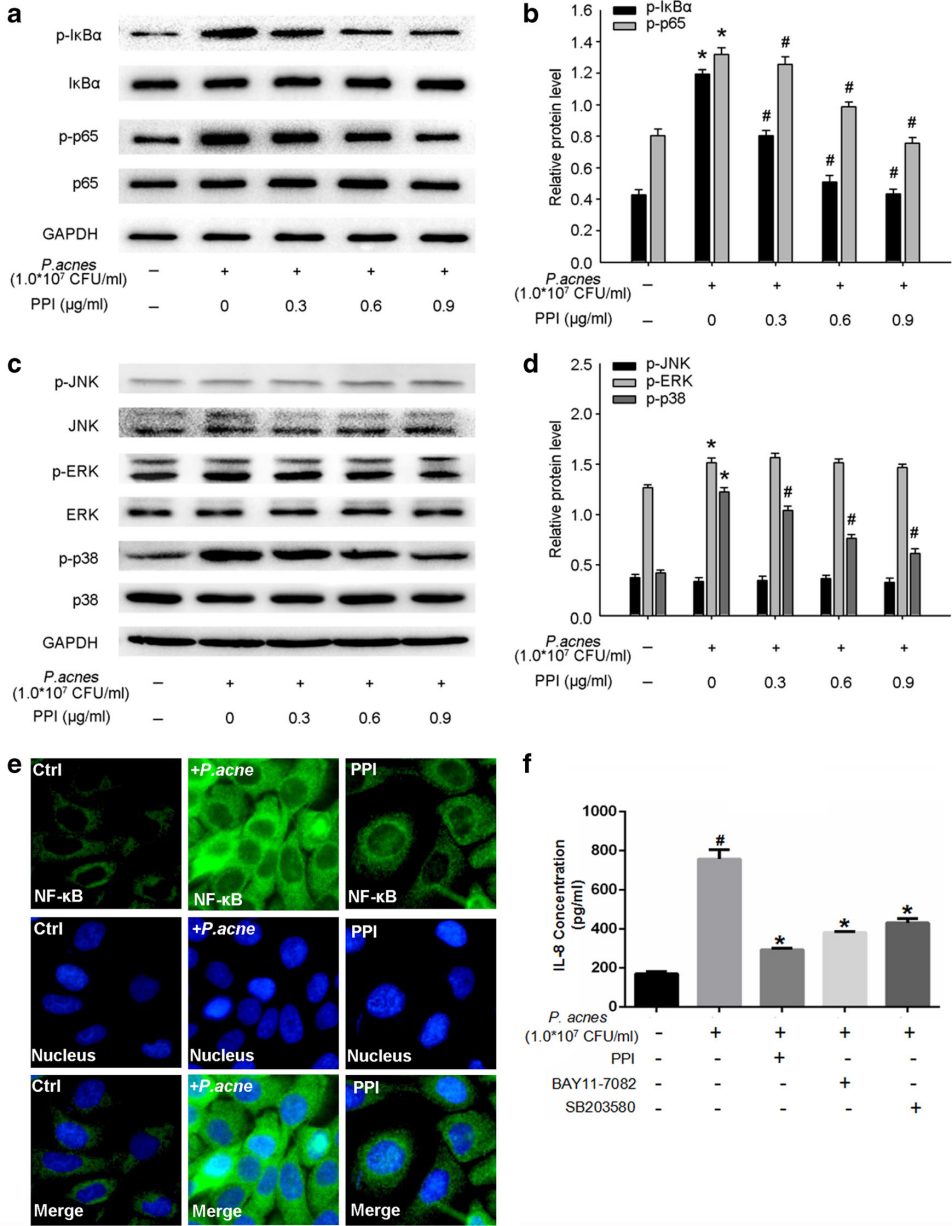
Effects of PPI on the NF-κB and MAPK signaling pathways in heat-killed *P. acnes*-treated HaCaT keratinocytes. Cells were pretreated with 0.3, 0.6, and 0.9 μg/ml of PPI for 2 h, followed by stimulation with *P. acnes* for 24 h. Expression of NF-κB (**a**, **b**) and MAPK (**c**, **d**) proteins was analyzed by western blotting, and the translocation of NF-κB p65 was detected by immunofluorescence (**e**). All data represent three independent experiments. *P < 0.05 between control and each *P. acnes*-stimulated group, #*P* < 0.05 between the *P. acnes*-treated group, PPI non-treated group, and each PPI-treated group. Heat-killed *P. acnes* (107 CFU/ml) induced the secretion of IL-8, which is suppressed by BAY11-7082 (10 μM), SB203580 (20 μM), and PPI (0.9 μg/ml) (**f**). *P < 0.05 between *P. acnes*-stimulated only and treated with BAY11-7082 (10 μM), SB203580 (20 μM), or PPI (0.9 μg/ml). #*P* < 0.05 between control and each *P. acnes*-stimulated group.

To investigate whether the NF-κB signaling pathway is involved in the anti-inflammatory properties of PPI, we examined the expression profile of NF-κB-related proteins by western blotting. We found that phosphorylated IκBα and NF-κB p65 levels were significantly upregulated after *P. acnes* treatment but were downregulated by co-treatment with PPI (Fig. 4a, b). We used immunofluorescence staining to examine the location of NF-κB p65 in HaCaT cells. We discovered that NF-κB p65-positive staining was predominately localized in control cytoplasm and shifted to the nuclei with *P. acnes* stimulation. Interestingly, the *P. acnes*-induced translocation of NF-κB p65 was suppressed in the presence of PPI (Fig. 4e).

MAPK activation is important in the regulation of the inflammatory response. The phosphorylation of p38 was significantly upregulated in HaCaT keratinocytes following *P. acnes* treatment, and ERK phosphorylation slightly increased (Fig. 4c, d). In contrast, PPI inhibited *P. acnes*-induced p38 phosphorylation in a concentration-dependent manner. However, the effect of PPI on ERK phosphorylation was not significant (Fig. 4c, d). Moreover, treatment with *P. acnes* and PPI did not have any effect on JNK phosphorylation (Fig. 4c, d). Additionally, the NF-κB p65 inhibitor (BAY11-7082), p38 MAPK inhibitor (SB203580), and PPI inhibited IL-8, TNF-α, and IL-6 expression in heat-killed *P. acnes*-treated cells (Fig. 4f and Supplemental Figs. 1 and 2).

## DISCUSSION

Acne vulgaris is a chronic inflammatory skin disorder that affects the pilosebaceous unit. Although the exact pathogenesis of acne vulgaris has not yet been elucidated, it has been verified that *P. acnes* colonization and proliferation play important roles in the development of acne inflammation [22, 30]. *P. acnes* promotes the activation of TLR2 in keratinocytes, aggravating inflammation reactions [14, 17]. In fact, TLR activation leads to activation of the MAPK and NF-κB signaling pathways [24]. MAPK activation is important for mediating the signaling cascades that regulate NF-κB activation [28, 32]. In addition, activation of both MAPK and NF-κB have been found in acne lesions, suggesting that the MAPK and NF-κB signaling pathways are important for the pathogenesis of acne vulgaris [18, 30]. Therefore, suppressing the activation of MAPK and NF-κB has a protective effect on acne inflammation.

PPI is an ethanol extract of *Paris polyphylla* Smith var. *yunnanensis* (Franch.) Hand.-Mazz., a natural herb that has been frequently used in traditional medicine for the treatment of hemostasis, snakebite, psoriasis, and abscesses for thousands of years [26]. Steroidal saponins, phytosterols, phytoecdysones, flavonoids, and fatty acid esters are the main components of *P. polyphylla* var. *yunnanensis* [31]. Among them, steroidal saponins such as polyphyllin I (PPI), II (PPII), VI (PPVI), and VII (PPVII) are the main active constituents [26, 31]. Studies have shown a direct effect of *P. acnes* on keratinocytes *via* interaction with TLR2 that induces the production of various inflammatory cytokines, thereby promoting acne inflammation [10]. Moreover, there is a positive correlation between the severity of acne inflammation and TLR2 expression levels [2]. Zinc salts and systemic isotretinoin are effective against inflammatory acne *via* reduction in TLR2 expression in human keratinocytes [2, 12]. Therefore, we analyzed PPI in subsequent experiments to investigate whether PPI inhibits the production of common inflammatory cytokines and the expression of TLR2 as well as its downstream signaling pathways.

In our current study, TLR2 mRNA and protein expression levels in HaCaT cells were significantly increased after *P. acnes* treatment; however, PPI suppressed TLR2 expression. Follicular epidermal dyskeratosis is one of the major triggers of acne pathogenesis. IL-1α induces hypercornification of the infundibulum in a manner similar to that seen in comedones [8, 21]. K16, a marker of epidermal proliferation and abnormal differentiation, is upregulated in all hyperproliferative and abnormally differentiating suprabasal keratinocytes [29]. We examined and quantified the changes in IL-1α and K16 through qRT-PCR and western blotting; the results demonstrated that IL-1α and K16 expression levels were significantly increased in HaCaT keratinocytes following *P. acnes* stimulation but were significantly downregulated by PPI. Therefore, PPI might reverse the altered keratinization of follicular keratinocytes by decreasing IL-1α levels.

The *P. acnes*-induced production of various common inflammatory cytokines represents a key pathogenic factor resulting in disease initiation and aggravation. Serum IL-6 levels are significantly higher in acne patients than in the normal population, suggesting a vital role for IL-6 in the pathogenesis of acne [20]. IL-8, a CXC chemokine with mitogenic activity in keratinocytes, is involved in the recruitment of neutrophils, the predominant cell type involved in acne inflammation-related skin lesions [16]. TNF-α is a multifunctional cytokine related to the regulation of acne inflammation [25]. According to our results, treatment with heat-killed *P. acnes* upregulated the expression levels of IL-6, IL-8, and TNF-α, with a particularly robust effect on IL-8, while PPI inhibited the production of these proinflammatory cytokines in a dose-dependent manner. Thus, PPI might inhibit the inflammatory cascade by suppressing the production of these inflammatory cytokines.

*P. acnes*-activated TLR2 triggers the activation of the NF-κB and MAPK signaling pathways, which are responsible for inflammatory cytokine production and the innate immune response [19, 24]. The levels of phosphorylated IκBα and NF-κB p65 were significantly upregulated after *P. acnes* treatment but were downregulated by PPI. There are three MAPKs: p38, ERK, and JNK. The phosphorylation of p38 was significantly increased, and ERK was slightly increased in HaCaT keratinocytes following *P. acnes* treatment. Interestingly, PPI inhibited the *P. acnes*-induced phosphorylation of p38 in a concentration-dependent manner but did not have any effect on ERK phosphorylation. Moreover, our results showed that treatment with *P. acnes* and PPI did not have any effect on JNK phosphorylation. Subsequently, treatment with a p38 MAPK inhibitor (SB203580) or PPI decreased IL-8 expression in heat-killed *P. acnes*-treated cells. These results demonstrated that PPI inhibits IL-8 expression, possibly by suppressing NF-κB associated protein and p38 MAPK phosphorylation in heat-killed *P. acnes*-stimulated HaCaT cells. IL-8 is commonly found at high levels in acne lesions and is involved in the recruitment of neutrophils, which are the predominant cell type in acne-related lesions [16]. Nicotinamide, the amide derivative of vitamin B3, has been shown to be clinically effective in the treatment of acne by inhibiting IL-8 production [6]. Therefore, we hypothesized that the anti-inflammatory effects of PPI might be at least partly attributable to the inhibition of IL-8 production.

In conclusion, we demonstrated that PPI has protective effects against *P. acnes*-induced inflammation and hypercornification, showing the potential clinical feasibility of PPI in acne treatment.

## Electronic supplementary material

Supplemental Figure 1 Cells were pretreated with BAY11–7082 (10 μM), SB203580 (20 μM) or PPI (0.9 μg/ml) for 2 h, followed by stimulating with *P.acnes* for 24 h. Expression of TNF-α was detected by ELISA (Fig. 1). *P < 0.05 between *P.acnes*-stimulated only and treated with BAY11–7082 (10 μM), SB203580 (20 μM) or PPI (0.9 μg/ml). #P < 0.05 between control and each *P.acnes*-stimulated group. (PNG 238 kb)

High resolution image (TIF 452 kb)

Supplemental Figure 2 Cells were pretreated with BAY11–7082 (10 μM), SB203580 (20 μM) or PPI (0.9 μg/ml) for 2 h, followed by stimulating with *P.acnes* for 24 h. Expression of IL-6 was detected by ELISA (Fig. 1). *P < 0.05 between *P.acnes*-stimulated only and treated with BAY11–7082 (10 μM), SB203580 (20 μM) or PPI (0.9 μg/ml). #P < 0.05 between control and each *P.acnes*-stimulated group. (PNG 189 kb)

High resolution image (TIF 346 kb)

Supplemental Figure 3 Cells were pretreated with BAY11–7082 (10 μM), SB203580 (20 μM) or PPI (0.9 μg/ml) for 2 h, followed by stimulating with *P.acnes* for 24 h. Expression of TLR2 was analyzed by western blotting. (PNG 107 kb)

High resolution image (TIF 172 kb)
