# Supplementary figures and images for: Polyphyllin I Inhibits Propionibacterium acnes-Induced Inflammation In Vitro
Source: Inflammation. 2018 Aug 18;42(1):35–44. doi: 10.1007/s10753-018-0870-z (PMC6394558; doi:10.1007/s10753-018-0870-z)

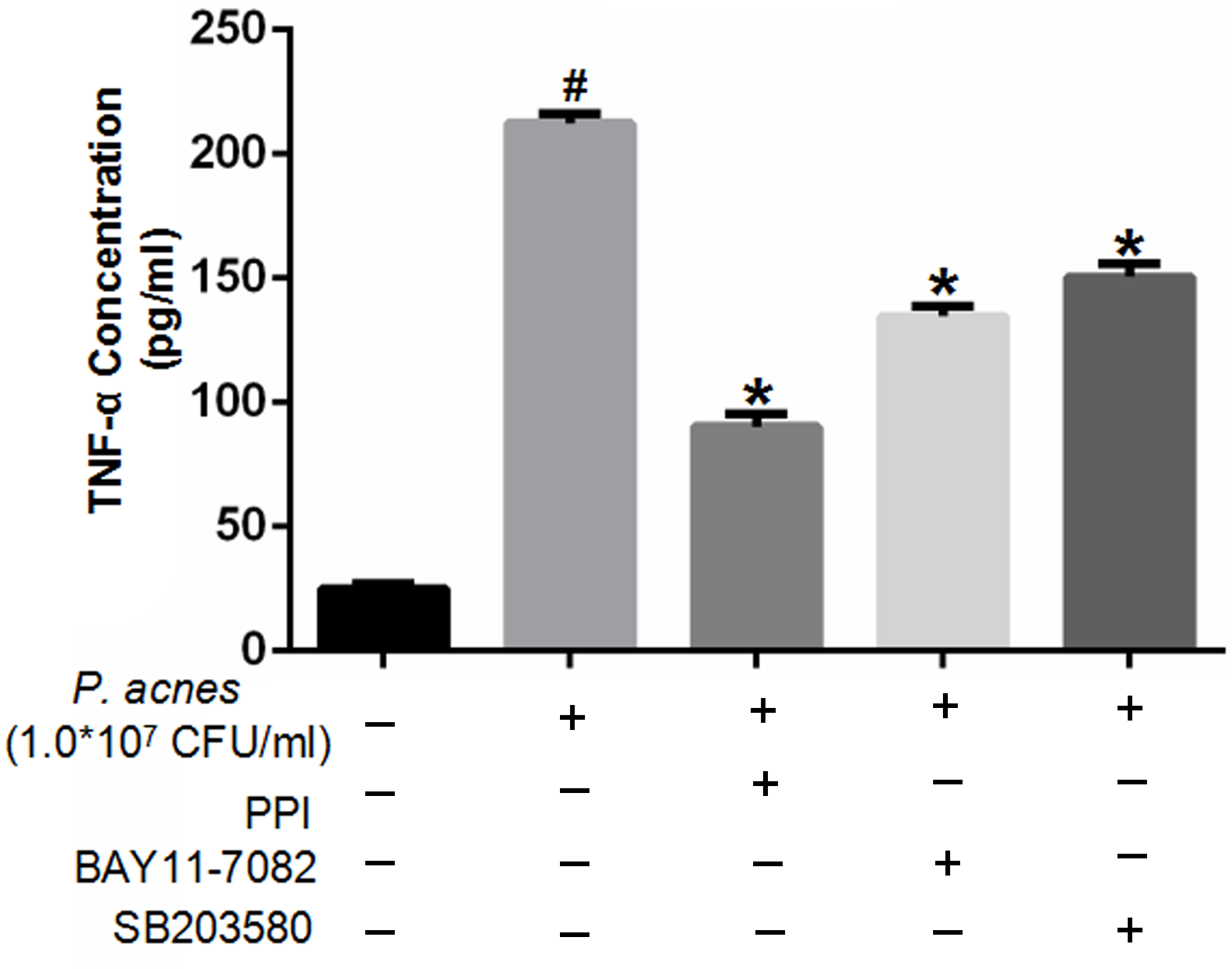

Supplement: Supplementary file 1 — Cells were pretreated with BAY11–7082 (10 μM), SB203580 (20 μM) or PPI (0.9 μg/ml) for 2 h, followed by stimulating with P.acnes for 24 h. Expression of TNF-α was detected by ELISA (Fig. 1). *P < 0.05 between P.acnes-stimulated only and treated with BAY11–7082 (10 μM), SB203580 (20 μM) or PPI (0.9 μg/ml). #P < 0.05 between control and each P.acnes-stimulated group. (PNG 238 kb) [file 10753_2018_870_Fig5_ESM.png]

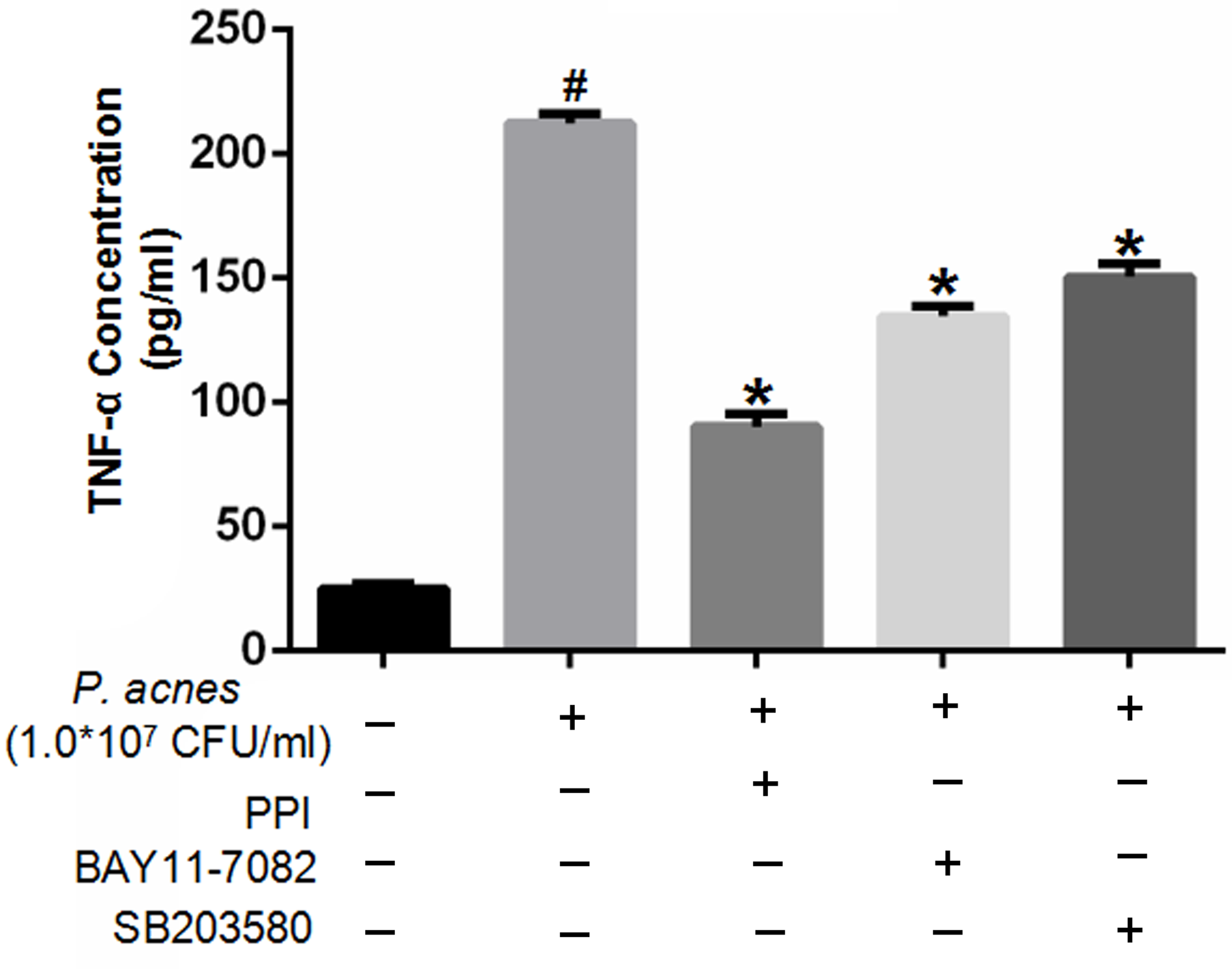

Supplement: Supplementary file 2 — High resolution image (TIF 452 kb) [file 10753_2018_870_MOESM1_ESM.tif]

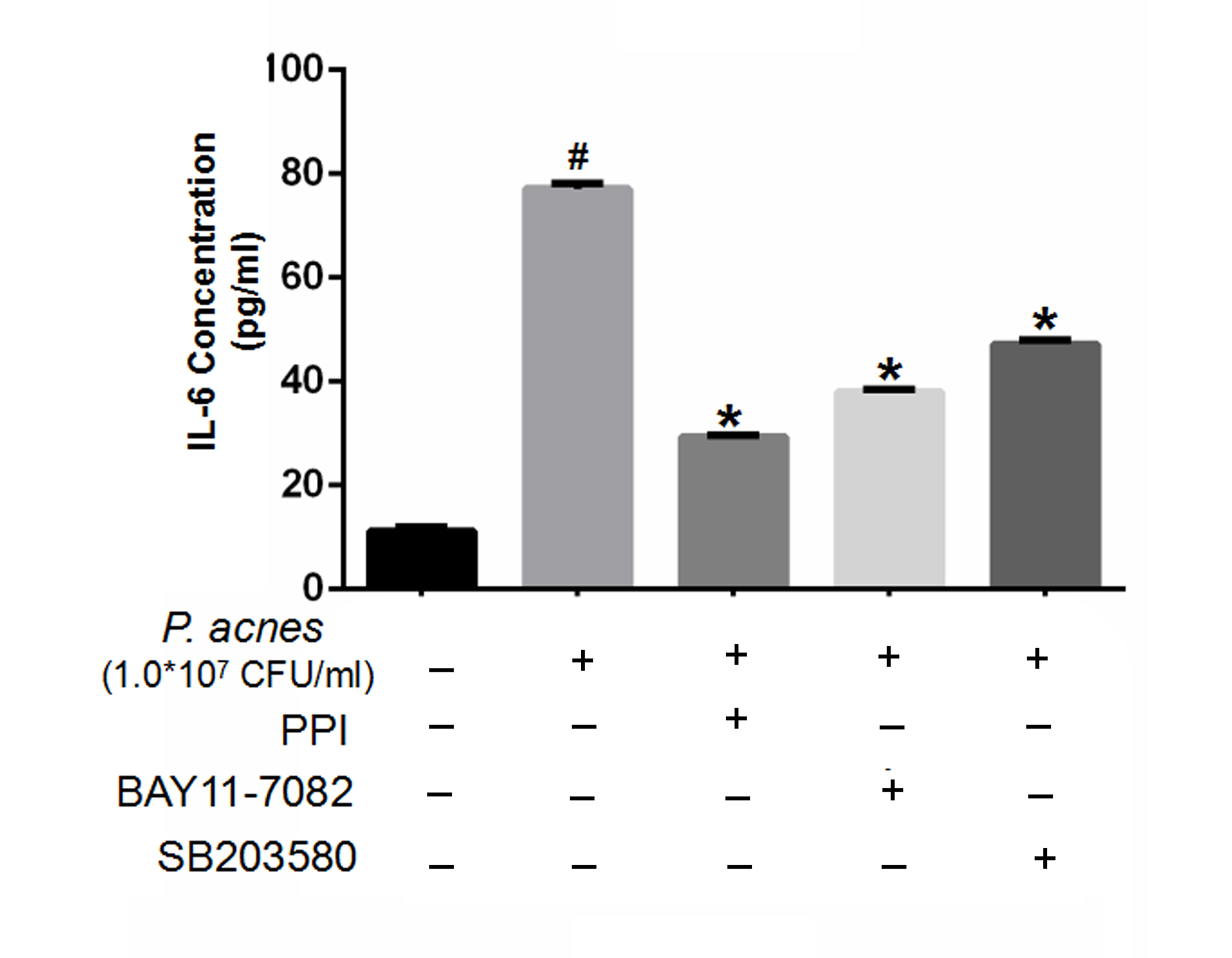

Supplement: Supplementary file 3 — Cells were pretreated with BAY11–7082 (10 μM), SB203580 (20 μM) or PPI (0.9 μg/ml) for 2 h, followed by stimulating with P.acnes for 24 h. Expression of IL-6 was detected by ELISA (Fig. 1). *P < 0.05 between P.acnes-stimulated only and treated with BAY11–7082 (10 μM), SB203580 (20 μM) or PPI (0.9 μg/ml). #P < 0.05 between control and each P.acnes-stimulated group. (PNG 189 kb) [file 10753_2018_870_Fig6_ESM.png]

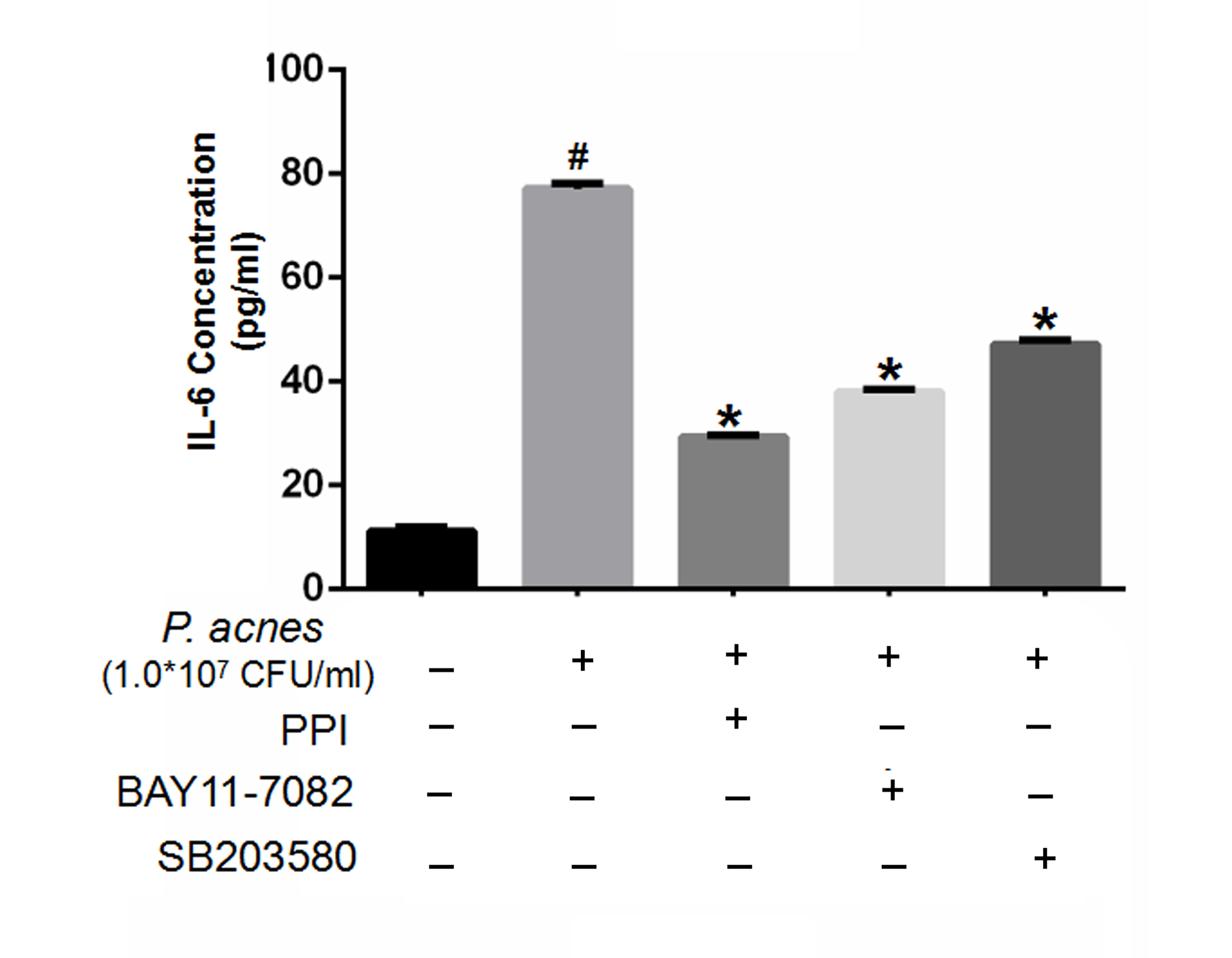

Supplement: Supplementary file 4 — High resolution image (TIF 346 kb) [file 10753_2018_870_MOESM2_ESM.tif]

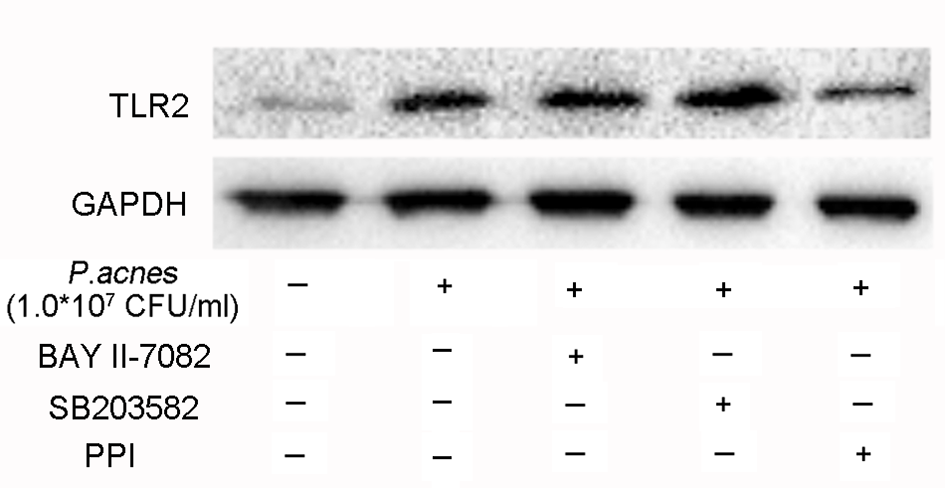

Supplement: Supplementary file 5 — Cells were pretreated with BAY11–7082 (10 μM), SB203580 (20 μM) or PPI (0.9 μg/ml) for 2 h, followed by stimulating with P.acnes for 24 h. Expression of TLR2 was analyzed by western blotting. (PNG 107 kb) [file 10753_2018_870_Fig7_ESM.png]

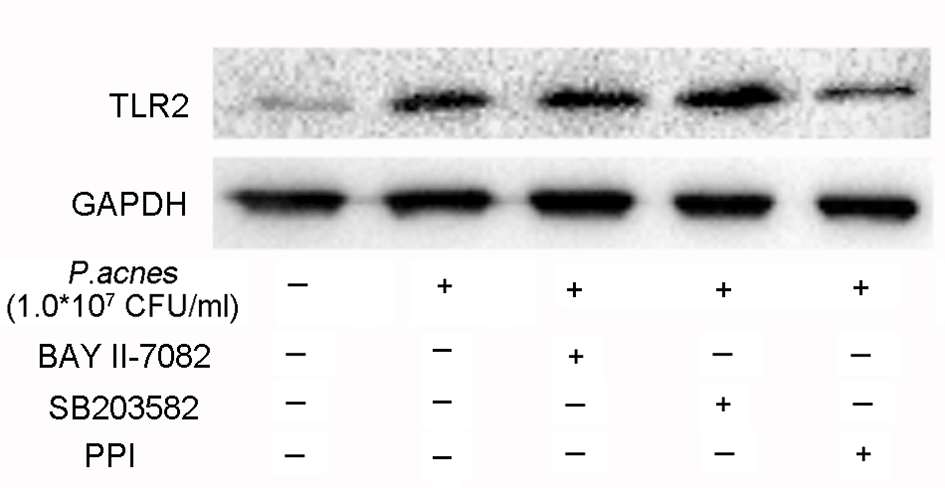

Supplement: Supplementary file 6 — High resolution image (TIF 172 kb) [file 10753_2018_870_MOESM3_ESM.tif]
